# Causal relationships of lifestyle behaviours and body fat distribution on diabetic microvascular complications: a Mendelian randomization study

**DOI:** 10.3389/fgene.2024.1381322

**Published:** 2024-07-08

**Authors:** Nuojin Guo, Hekai Shi, Hongmei Zhao, Yierfan Abuduani, Da Chen, Xishuang Chen, Hua Wang, Peicheng Li

**Affiliations:** ^1^ Department of Endocrinology, Shanghai East Hospital, Tongji University School of Medicine, Shanghai, China; ^2^ Department of Bariatric and Metabolic Surgery, Fudan University Affiliated Huadong Hospital, Shanghai, China

**Keywords:** lifestyle behaviours, body fat distribution, diabetic microvascular complications, causality, Mendelian randomization study

## Abstract

**Objectives:**

To determine the causal correlations of lifestyle behaviours and body fat distribution on diabetic microvascular complications through a Mendelian Randomization (MR).

**Methods:**

Genetic variants significantly associated with lifestyle behaviours, abdominal obesity, generalized obesity and diabetic microvascular complications were extracted from the UK Biobank (UKB) and FinnGen. The inverse variance weighted (IVW) method was regarded as the primary method. The main results were presented in odds ratio (OR) per standard deviation (SD) increase, and a series of sensitivity analyses were also conducted to validate the stability of the results.

**Results:**

There was a positive causal correlation between smoking and the development of diabetic retinopathy (OR = 1.16; 95%CI: 1.04–1.30; *p* = 0.01). All of the indicators representing abdominal obesity had a statistically significant causal association with diabetic microvascular complications. Concerning generalized obesity, there were significant causal associations of body mass index (BMI) on diabetic nephropathy (OR = 1.92; 95%CI: 1.58–2.33; *p* < 0.001), diabetic retinopathy (OR = 1.27; 95%CI: 1.15–1.40; *p* < 0.001), and diabetic neuropathy (OR = 2.60; 95%CI: 1.95–3.45; *p* < 0.001). Other indicators including leg fat mass (left), and arm fat mass (left) also had a significant positive causality with diabetic microvascular complications.

**Conclusion:**

Our findings suggested that smoking has a genetically causal association with the development of diabetic retinopathy rather than diabetic nephropathy and diabetic neuropathy. In addition, both abdominal obesity and generalized obesity are risk factors for diabetic microvascular complications. To note, abdominal obesity represented by waist circumference (WC) is the most significant risk factor.

## 1 Introduction

Diabetes mellitus (DM) is a metabolic disease caused by genetic and environmental factors. The International Diabetes Federation (IDF) has listed DM as one of the fastest-developing diseases in this century. In the past 3 decades, the amount of DM patients has quadrupled worldwide, which is expected to increase to 578 million by 2030 (1). The impairment of the function of pancreatic β cells leads to abnormal blood glucose levels (Rachdaoui, 2020). Long-term exposure to a high glucose environment could destroy macrovascular and microvascular systems and contribute to complications such as diabetic nephropathy, diabetic retinopathy, and diabetic neuropathy. A healthy lifestyle plays an extremely significant role in the prevention and treatment of diabetes (Martín-Peláez et al., 2020). Studies have suggested that different lifestyles, including smoking, physical activity, alcohol intake, overweight, and diet quality could affect life expectancy and the incidence of chronic diseases such as diabetes and the corresponding microvascular complications (Loef and Walach, 2012; Li et al., 2018; Li et al., 2020). In addition, it’s been reported that among young people, unhealthy lifestyles such as obesity and sedentary habits are the main factors inducing type 2 diabetes, and the earlier diabetes is diagnosed, the more patients are prone to suffer from long-term complications. The extent of insulin resistance is directly related to severe obesity, especially excess visceral adiposity. Recent studies using high-throughput metabolomics techniques to evaluate metabolite markers have also shown that adipose tissue from obese insulin resistance and T2DM individuals exhibited unique lipidomic characteristics, which were associated with an increased risk of insulin resistance (Al-Sulaiti et al., 2018; Al-Sulaiti et al., 2019; Lascar et al., 2018). It’s noteworthy that obesity is considered to be a heterogeneous condition. People with similar body mass index (BMI) but diverse body fat distribution may have distinct metabolic and chronic disease risk characteristics (Goossens, 2017; Piché et al., 2018). Therefore, further surrogate indicators of body fat distribution are urgently needed in the evaluation of diabetic microvascular complications.

Increasing observational studies and meta-analyses have embarked on exploring the association between unhealthy lifestyle behaviours and diabetic microvascular complications (Uusitupa et al., 2019; Chudasama and Khunti, 2023; Geng et al., 2023; Liu et al., 2023). Liu et al. have found that adhering to an overall healthy lifestyle, which includes high-quality diets, moderate alcohol consumption, non-smoking, moderate to vigorous physical activity, and a healthy weight is associated with a significantly lower risk of microvascular complications among diabetes patients (Liu et al., 2023). Geng et al. further demonstrated that blood lipid-related biomarkers such as apolipoprotein A, high-density lipoprotein, and triglycerides could affect the association between lifestyle and diabetic microvascular complications (Geng et al., 2023). However, potential confounding resulting from genetic susceptibility, psychological stress, or medication therapy cannot be eliminated. However, most of the relevant researches are cross-sectional and retrospective cohorts, which couldn’t prove causality because they were essentially observational.

Mendelian randomization (MR) analysis is conducted regarding single-nucleotide polymorphisms (SNPs) as instrumental variables (IVs) to explore the causal association between exposure and clinically relevant outcomes. The alleles of this exposure-related genetic variation are randomly assigned and are not affected by potential confounding or reverse causality (Sekula et al., 2016; Bowden and Holmes, 2019). We aim to comprehensively assess the associations between diverse lifestyle behaviours such as smoking, moderate to vigorous physical activity, alcohol intake frequency and body fat distribution indicators such as BMI, waist circumference (WC), and body fat percentage with diabetic microvascular complications genetically in order to provide more references to identify and evaluate high-risk individuals with diabetic microvascular complications.

## 2 Materials and methods

### 2.1 Study design

**FIGURE 1:**
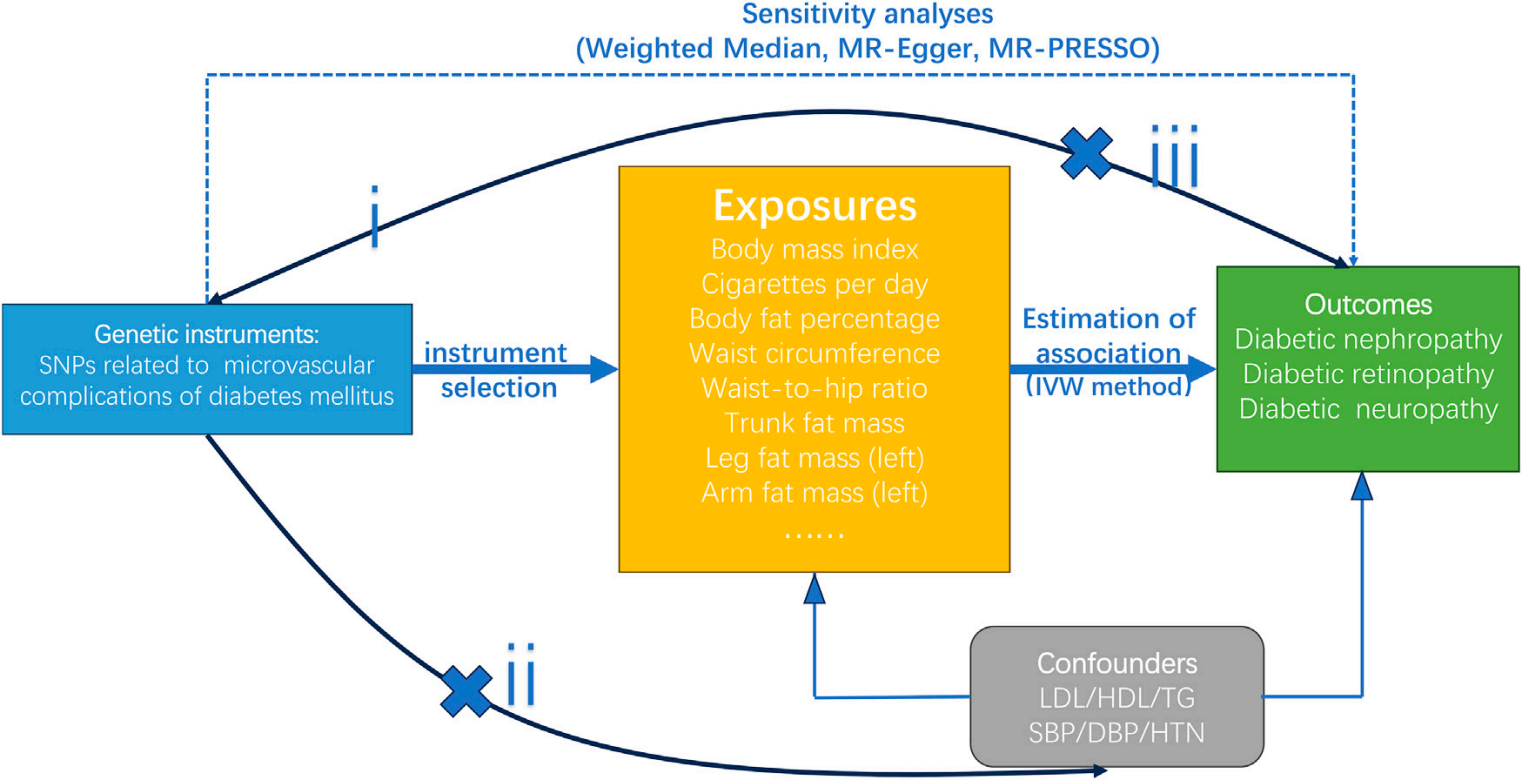
Principle diagram of the MR assumption: (i) the genetic variants are associated with the risk factors; (ii) the genetic variants aren’t correlated with confounders; (iii) the genetic variants influence the outcome only via the risk factors.

We conducted an MR analysis to assess whether lifestyle behaviours (i.e., the intensity of physical activity, smoking, alcohol intake, and sleep), and body fat distribution are genetically associated with diabetic microvascular complications. To note, diabetic nephropathy, diabetic retinopathy, and diabetic neuropathy were selected to represent diabetic microvascular complications in this study. The abdominal obesity was assessed by WC, waist-to-hip ratio (WHR), trunk fat mass (TFM), and hip circumference (HC). The generalized obesity was evaluated by BMI, body fat percentage, leg fat mass, and arm fat mass. All of the largest publicly available GWASs were obtained from the UK Biobank (UKB) and FinnGen. This MR analysis was based on three assumptions (Izadi et al., 2022): the genetic variants are associated with the risk factors (Rachdaoui, 2020); the genetic variants aren’t correlated with confounders (Martín-Peláez et al., 2020); the genetic variants influence the outcome only via the risk factors (Boef et al., 2015). The principle graph is presented in Figure 1. Only genetic variants with genome-wide significance (*p* < 5 × 10^−8^) were extracted as IVs, and the level of the *p*-value would be reduced to 5 × 10^−6^ if the number of SNPs extracted was less than 10. In addition, R^2^ and F values were calculated according to the formula mentioned in the previous articles to detect weak IVs (Sekula et al., 2016; Bowden and Holmes, 2019). We screened SNPs (R^2^ < 0.001) to eliminate linkage disequilibrium. SNPs with an F value less than 10 would be considered weak IVs and removed. Finally, we searched the PhenoScanner website: http://www.phenoscanner.medschl.cam.ac.uk/ to filter for all traits or diseases associated with outcome diseases and removed corresponding SNPs with confounding factors.

### 2.2 Data source

In this study, the intensity of physical activity, smoking, alcohol intake, and sleep were selected as representative lifestyle behaviours. Genetic instrumental variants significantly associated with cigarettes per day were extracted from the consortium of GWAS and Sequencing Consortium of Alcohol and Nicotine use (GWAS ID: ieu-b-25). Genetic instrumental variables significantly associated with moderate to vigorous physical activity levels were extracted from UKB (GWAS ID: ebi-a-GCST006097). Other GWAS datasets including alcohol intake frequency, sleeplessness/insomnia, and sleep duration were extracted from the Medical Research Council Integrative Epidemiology Unit (MRC-IEU) consortium with sample sizes of more than 400,000. Genetic instrumental variants significantly associated with BMI and WHR were extracted from the UKB. Genetic instrumental variants significantly associated with HC, TFM, WC, leg fat mass (left), and leg fat mass (left) were extracted from the MRC-IEU consortium. The genetic instrumental variants significantly associated with diabetic microvascular complications were all from FinnGen with huge sample sizes of over 200,000. Detailed information was provided in Table 1.

**TABLE 1:** Overview of the summary datasets.

Characteristic		Resource	Sample size	Number of SNPs	Population	Year	GWAS ID
Exposure							
Physical activity	Moderate to vigorous physical activity levels	UK Biobank	377,234	11,808,007	European	2018	ebi-a-GCST006097
Smoke	Cigarettes per day	UK Biobank	337,334	11,913,712	European	2019	ieu-b-25
Alcohol intake	Alcohol intake frequency	UK Biobank	462,346	9,851,867	European	2018	ukb-b-5779
Body fat distribution	Body mass index	UK Biobank	457,756	4,238,669	European	2021	ebi-a-GCST90025994
	Body fat percentage	UK Biobank	454,633	9,851,867	European	2018	ukb-b-8909
	Hip circumference	UK Biobank	462,117	9,851,867	European	2018	ukb-b-15590
	Waist circumference	UK Biobank	462,166	9,851,867	European	2018	ukb-b-9405
	Waist-hip ratio	UK Biobank	502,773	11,973,122	European	2018	ebi-a-GCST90029009
	Trunk fat mass	UK Biobank	454,588	9,851,867	European	2018	ukb-b-20044
	Leg fat mass (left)	UK Biobank	454,823	9,851,867	European	2018	ukb-b-7212
	Arm fat mass (left)	UK Biobank	454,684	9,851,867	European	2018	ukb-b-8338
Sleep	Sleeplessness/ insomnia	UK Biobank	462,341	9,851,867	European	2018	ukb-b-3957
	Sleep duration	UK Biobank	460,099	9,851,867	European	2018	ukb-b-4424
Outcome							
Diabetic nephropathy		FinnGen	ncase: 3,283 ncontrol: 181,704	16,380,336	European	2021	finn-b-DM_NEPHROPATHY_EXMORE
Diabetic retinopathy		FinnGen	ncase: 14,584 ncontrol: 202,082	16,380,459	European	2021	finn-b-DM_RETINOPATHY
Diabetic neuropathy		FinnGen	ncase: 1,415ncontrol: 162,201	16,380,195	European	2021	finn-b-DM_NEUROPATHY

GWAS, genome-wide association study; SNP, Single Nucleotide Polymorphism.

All of the populations contained in both the exposure and outcome datasets were of European ancestry from different countries and there was no large-scale overlap between participants that were included in GWAS datasets of exposure and outcome mentioned above. In addition, all of the GWAS datasets were publicly available. Therefore, ethical approval is not required accordingly.

### 2.3 Statistical analyses

After the extraction of data and harmonization of the effect alleles across GWASs, we conducted three tests for causal estimation. IVW method is the main method applied in this study, which pooled every single Wald estimate for each SNP and showed an accurate estimate of causal effects when all IVs met three assumptions (Burgess et al., 2013). The weighted median method could provide unbiased pooled results even if up to 50% of the IVs were invalid (Bowden et al., 2016). The MR-Egger method could help to identify and correct any bias introduced by horizontal pleiotropy (Burgess and Thompson, 2017). When the *p*-value of the IVW method is less than 0.05 and the results obtained by more than two methods have the same direction, the causality can be considered nominally significant. The MR-Egger intercept was conducted to test horizontal pleiotropy. If the *p*-value was less than 0.05, the causality couldn’t be validated. To further check for the validity and stability of the MR results, the sensitivity analysis was subsequently conducted using the “leave-one-out”, drawing funnel plots and the Mendelian Randomization Pleiotropy Residual Sum and Outlier (MR-PRESSO), which could assess whether the exclusion of potential outlier SNPs influences the results (Burgess et al., 2013). To quantify heterogeneity in the SNP effects across studies, we calculated Cochran’s IVW Q statistics, and corresponding *p* values. If the *p*-value was less than 0.05, the heterogeneity existed Greco et al., 2015. Furthermore, we calculated the statistical power of this MR analysis via the following website: https://shiny.cnsgenomics.com/mRnd/. The results were shown as odds ratio (OR) per standard deviation (SD) increase with 95% confidence intervals (95% CI). *p* < 0.05 (two-tailed) was considered statistically significant. All statistical analyses were performed in R (version 4.1.3) using the R package “Two sample MR” (version 0.5.7) and “MRPRESSO” (version 1.0).

## 3 Results

### 3.1 Association between lifestyle behaviours and diabetic microvascular complications

**FIGURE 2:**
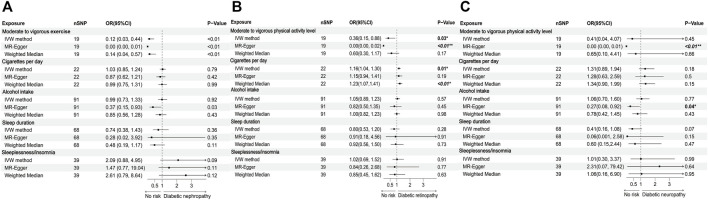
Forest plots of lifestyle behaviours associated with the risk of **(A)** diabetic nephropathy, **(B)** diabetic retinopathy, and **(C)** diabetic neuropathy.

19 SNPs associated with moderate to vigorous physical activity levels at genome-wide significance were identified, and no weak IVs were excluded. IVW method indicated that there was a negative causal correlation between moderate to vigorous physical activity levels and diabetic nephropathy (OR = 0.12; 95%CI: 0.03–0.44; *p* = 0.001) and diabetic retinopathy (OR = 0.36; 95%CI: 0.15–0.88; *p* = 0.03). However, the MR-Egger intercept analysis indicated that horizontal pleiotropy existed (*p* = 0.002 and 0.0007 respectively). Therefore, the causality couldn’t be validated. 22 SNPs associated with smoking at genome-wide significance were selected, and the IVW method demonstrated that there was a positive causal correlation between smoking and the development of diabetic retinopathy (OR = 1.16; 95%CI: 1.04–1.30; *p* = 0.01). However, there was no statistically significant association between smoking and diabetic nephropathy (OR = 1.03; 95%CI: 0.85–1.24; *p* = 0.79), or diabetic neuropathy (OR = 1.31; 95%CI: 0.89–1.94; *p* = 0.18). 91 SNPs strongly associated with alcohol intake frequency were extracted, with no weak IVs removed. IVW method demonstrated that there was no statistically significant causal correlation between alcohol intake frequency and diabetic microvascular complications. 68 SNPs strongly associated with sleep duration and 39 SNPs strongly associated with insomnia were selected. We found that both sleep duration and insomnia were not markedly associated with diabetic microvascular complications. All of the analysis results mentioned above were shown in Figure 2, and the specific *p* values were listed in Supplementary Tables S1–S3 (Supplementary Material).

### 3.2 Association between body fat distribution and diabetic microvascular complications

**FIGURE 3:**
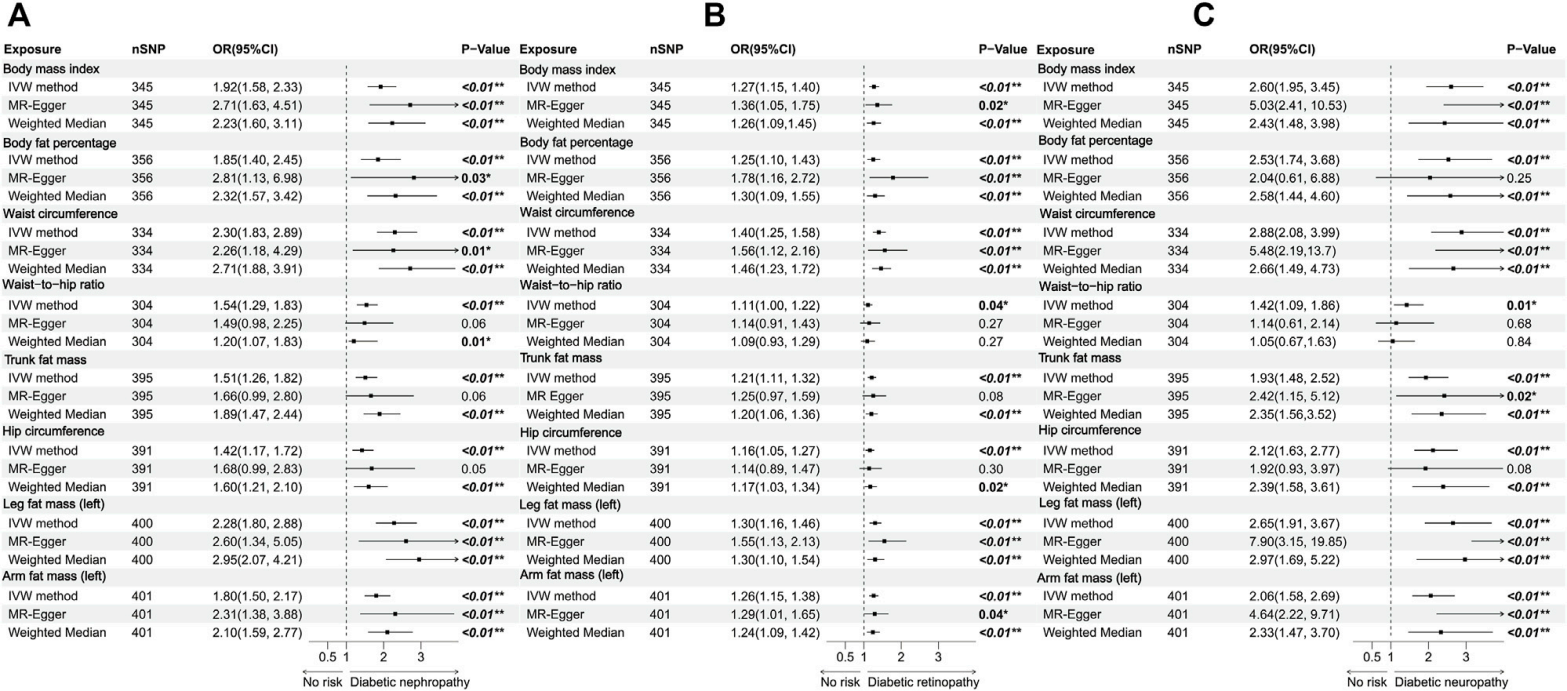
Forest plots of body fat distribution measurements associated with the risk of **(A)** diabetic nephropathy, **(B)** diabetic retinopathy, and **(C)** diabetic neuropathy.

Our MR analysis indicated that all of the indicators representing abdominal obesity, including WC, WHR, TFM, and HC had a statistically significant causal association with three kinds of diabetic microvascular complications. Concerning generalized obesity, there were significant causal associations of genetically predictive BMI on diabetic nephropathy (OR = 1.92; 95%CI: 1.58–2.33; *p* < 0.001), diabetic retinopathy (OR = 1.27; 95%CI: 1.15–1.40; *p* < 0.001), and diabetic neuropathy (OR = 2.60; 95%CI: 1.95–3.45; *p* < 0.001). 356 SNPs strongly associated with body fat percentage were extracted. IVW method demonstrated that there was a statistically significant causal correlation between body fat percentage and all of the three diabetic microvascular complications. Other generalized obesity measures including leg fat mass (left), and arm fat mass (left) were also significantly associated with diabetic nephropathy and retinopathy. To note, the MR-Egger intercept method suggested that horizontal pleiotropy existed between leg fat mass (left), arm fat mass (left), and diabetic neuropathy (*p* = 0.01 and 0.02 respectively). Therefore, the causality was proven invalid. All of the analysis results mentioned above were shown in Figure 3. Most of the adiposity-related exposures presented high statistical power (Supplementary Tables S4–S6; Supplementary Material).

### 3.3 Sensitivity analysis

In MR analysis, a series of methods were used to assess whether there was significant heterogeneity in the study. Partial pooled results had heterogeneity (Q value <0.05). However, the “leave-one-out” and MR-PRESSO methods presented good stability. The funnel plot illustrated the symmetric distribution of a single IV. Supplementary Figures S1–S6 (Supplementary Material) provided detailed information on the funnel plots and MR-Egger intercept scatter plots.

## 4 Discussion

Several observational studies have explored the association between changeable lifestyle behaviours and diabetic microvascular complications. The associations between smoking and diabetic nephropathy and neuropathy have been particularly widely documented. (Blomster et al., 2014; Dunkler et al., 2015; Diabetes Prevention Program Research Group., 2015; Kristensen et al., 2023). The mechanisms of how smoking impairs renal and neurological function are still not fully understood. It’s reported that heavy metals such as lead and chromium in tobacco accumulate in the blood, which thickens the glomerular basement membrane and damages the glomerulus (Cooper, 2006; Sliwińska-Mossoń et al., 2013). However, several previous studies pointed out that smoking was associated with oxidative stress and endothelial dysfunction independent of diabetes (Benowitz, 2003; Burke and Fitzgerald, 2003). Clair et al. conducted one large meta-analysis including 38 studies (10 prospective cohorts and 28 cross-sectional), which suggested that individuals with type 2 diabetes showed no statistically significant association between smoking and diabetic neuropathy (Clair et al., 2015). Diabetes Control and Complications Trial/Epidemiology of Diabetes Interventions and Complications (DCCT/EDIC) Study in 2020 also did not establish smoking as a major risk factor for diabetic neuropathy (Braffett et al., 2020). We reckoned the potential reasons for the heterogeneity might be that most of the relevant clinical studies are cross-sectional and retrospective cohorts due to multifactorial pathogenesis and long follow-up periods, which couldn’t prove causality because they were essentially observational. Traditional observational studies might produce biases on account of small sample sizes and potential confounding factors. In recent years, researchers also embarked on verifying the genetic causality between unhealthy lifestyle behaviours and diabetic retinopathy via MR analyses (Su et al., 2022). We performed this MR analysis to comprehensively figure out the causal correlations of the intensity of physical activity, smoking, alcohol intake, and sleeping on diabetic microvascular complications. Our study demonstrated a genetically causal association between smoking and diabetic retinopathy, while no causality was found among other included lifestyle behaviours and diabetic microvascular complications. We contained large-scale GWAS data, and SNPs strongly associated with exposures, which made the results more reliable.

Lipotoxicity is characterized by lipid accumulation in organs other than adipose tissue, which is mainly related to signal transduction dysfunction and insulin resistance in non-adipose tissues, such as the liver, kidney, myocardium, and pancreas (Opazo-Ríos et al., 2020). Abnormal accumulation of blood lipids mainly causes kidney injury-related diseases, especially diabetic nephropathy, by activating inflammation and oxidative stress, thus further promoting cell death (Vaziri, 2016). Several studies have indicated that obesity was a significant risk factor for developing diabetic nephropathy and diabetic retinopathy (Alicic et al., 2017; Lu et al., 2022; Zheng et al., 2023). However, some previous studies applied mere BMI and weight to assess obesity, which is lack of accuracy. On the one hand, BMI cannot distinguish between muscle mass and body fat. In this case, muscular people might have higher BMI and be misclassified as overweight or obese, while recent studies have demonstrated that greater muscle strength was significantly associated with lower mortality in patients with type 2 diabetes (Hamasaki et al., 2017). On the other hand, BMI cannot differentiate body fat distribution, therefore diabetic patients with excess visceral fat could be mistaken for normal (Han and Boyko, 2018). Furthermore, an unexpected hypothesis came into sight in recent years known as the “obesity paradox,” which suggested that obesity has a protective effect on mortality and cardiovascular disease in diabetic patients (Simati et al., 2023). The “obesity paradox” precisely embodies the incomplete assessment of obesity. Therefore, it’s reasonable to involve waist circumference, waist-to-hip ratio, trunk fat mass, hip circumference, body mass index, body fat percentage, leg fat mass, and arm fat mass. Our study indicated that both abdominal obesity and generalized obesity were significantly associated with diabetic microvascular complications. In addition, the increase in WC had a more significant effect on the pathogenesis of all of these three complications, which indicated that abdominal obesity is more closely related to diabetic microvascular complications than generalized obesity.

This study has several strengths. First, to our knowledge, this study is the first to comprehensively explore the causal relationship between lifestyle-related factors, different body fat distribution, and the risk of diabetic microvascular complications using the MR approach and the latest available large GWAS datasets. Second, given the high statistical power of most exposures and the consistency of the estimates using different MR approaches, our study was more convincing compared to conventional observational studies without potential confounding factors. Third, we managed to select indicators with the most significant causal association of diabetic microvascular complications. However, this MR analysis still had some limitations. First, all of the SNP corresponding study population were Europeans, which might lead to insufficient accuracy in other regions and races. Second, although the FinnGen covered huge sample sizes of over 200,000, sex, type of diabetes, and the duration of illness of the subjects weren’t provided in diabetic microvascular complication datasets. Therefore, more large-scale prospective studies are urgently needed for quantitative confirmation of such causality in the future. In addition, there were still some correlated heterogeneity and horizontal pleiotropy hard to eliminate.

## 5 Conclusion

This MR analysis demonstrates that smoking has a genetically positive association with the development of diabetic retinopathy. In addition, both abdominal obesity and generalized obesity are risk factors for diabetic microvascular complications. To note, abdominal obesity represented by WC is the most significant risk factor. For public health guidelines and clinical practice, our study highlights the significance of lifestyle management for patients with diabetes and provides a reference to identify high-risk individuals for diabetic microvascular complications.

## Data Availability

The original contributions presented in the study are included in the article/Supplementary Material, further inquiries can be directed to the corresponding authors.
