# Supplementary material for: Causal relationships of lifestyle behaviours and body fat distribution on diabetic microvascular complications: a Mendelian randomization study
Source: Front Genet. 2024 Jul 8;15:1381322. doi: 10.3389/fgene.2024.1381322 (PMC11264240; doi:10.3389/fgene.2024.1381322)
Supplement: Supplementary file 1 [file Table1.DOCX]

**Table S1. Mendelian randomization estimates of the associations between lifestyle behaviors, and body fat distribution and risk of diabetic nephropathy**

| Exposure | IVW method | | | MR-Egger | | Weighted Median | |
| --- | --- | --- | --- | --- | --- | --- | --- |
|  | OR (95%CI) | *P* Value | Cochran’s Q  (*P* Value) | OR (95%CI) | *P* Value | OR (95%CI) | *P* Value |
| Exercise | | | | | | | |
| Moderate to vigorous physical activity level | 0.12(0.03, 0.44) | **0.001*** | 40.17  (**0.002***) | 6.70e^-05^(1.02e^-06^, 0.004) | **0.0003*** | 0.14(0.04, 0.57) | **0.006*** |
| Smoke | | | | | | | |
| Cigarettes per day | 1.03(0.85, 1.24) | 0.79 | 18.76  (0.6) | 0.87(0.62, 1.21) | 0.42 | 0.99(0.75, 1.31) | 0.99 |
| Alcohol intake | | | | | | | |
| Alcohol intake frequency | 0.99(0.73, 1.33) | 0.92 | 120.46  (**0.02***) | 0.37(0.15, 0.93) | **0.03*** | 0.85(0.56, 1.28) | 0.43 |
| Sleep | | | | | | | |
| Sleep duration | 0.74(0.38, 1.43) | 0.36 | 81.01  (0.12) | 0.28(0.02, 3.92) | 0.35 | 0.48(0.19, 1.17) | 0.11 |
| Sleepless/  Insomnia | 2.09(0.88, 4.95) | 0.09 | 44.34  (0.22) | 1.47(0.11,19.04) | 0.77 | 2.61(0.79, 8.64) | 0.12 |
| Total adiposity | | | | | | | |
| Body mass index | 1.92(1.58, 2.33) | **7.52e^-11^*** | 443.7  (**0.0002***) | 2.71(1.63, 4.51) | **1.46e^-04^*** | 2.23(1.60, 3.11) | **1.97e^-06^*** |
| Body fat percentage | 1.85(1.40, 2.45) | **1.65e^-05^*** | 536.55  (**1.83e-09***) | 2.81(1.13, 6.98) | **0.03*** | 2.32(1.57, 3.42) | **2.44e^-05^*** |
| Body adiposity distribution | | | | | | | |
| Waist circumference | 2.30(1.83, 2.89) | **6.72e^-13^*** | 421.48  (**0.0007***) | 2.26(1.18, 4.29) | **0.01*** | 2.71(1.88, 3.91) | **8.832e^-08^*** |
| Waist-to-hip ratio | 1.54(1.29, 1.83) | **1.82e^-06^*** | 335.36  (0.09) | 1.49(0.98, 2.25) | 0.06 | 1.20(1.07, 1.83) | **0.01*** |
| Trunk fat mass | 1.51(1.26, 1.82) | **1.28e^-05^*** | 523.70  (**1.24e^-05*^**) | 1.66(0.99, 2.80) | 0.06 | 1.89(1.47, 2.44) | **9.01e^-07^*** |
| Hip circumference | 1.42(1.17, 1.72) | **3.35e^-04^*** | 571.01  (**2.20e^-08^***) | 1.68(0.99, 2.83) | 0.05 | 1.60(1.21, 2.10) | **8.80e^-04^*** |
| Leg fat mass (left) | 2.28(1.80, 2.88) | **4.67e^-12^*** | 518.69  (**4.85e^-05*^**) | 2.60(1.34, 5.05) | **0.005*** | 2.95(2.07, 4.21) | **2.12e^-09^*** |
| Arm fat mass (left) | 1.80(1.50, 2.17) | **5.52e^-10^*** | 519.48  (**5.06e^-05*^**) | 2.31(1.38, 3.88) | **0.002*** | 2.10(1.59, 2.77) | **1.54e^-07^*** |
| IVW, inverse‐variance weighted; OR, odds ratio; CI, confidence interval | | | | | | | |

**Table S2. Mendelian randomization estimates of the associations between lifestyle behaviors, and body fat distribution and risk of diabetic retinopathy**

| Exposure | IVW method | | | MR-Egger | | Weighted Median | |
| --- | --- | --- | --- | --- | --- | --- | --- |
|  | OR (95%CI) | *P* Value | Cochran’s Q  (P Value) | OR (95%CI) | *P* Value | OR (95%CI) | *P* Value |
| Exercise | | | | | | | |
| Moderate to vigorous physical activity level | 0.36(0.15, 0.88) | **0.03*** | 77.73  **(2.13e^-09*^**) | 0.001(8.621154e^-05^, 0.02) | **1.95e^-04^*** | 0.60(0.30, 1.17) | 0.17 |
| Smoke | | | | | | | |
| Cigarettes per day | 1.16(1.04, 1.30) | **0.01*** | 28.95  (0.12) | 1.15(0.94, 1.41) | 0.19 | 1.23(1.07,1.41) | **0.003*** |
| Alcohol intake | | | | | | | |
| Alcohol intake frequency | 1.05(0.89, 1.23) | 0.57 | 140.26  (**0.0005***) | 0.82(0.50,1.35) | 0.45 | 1.00(0.82, 1.23) | 0.98 |
| Sleep | | | | | | | |
| Sleep duration | 0.80(0.53, 1.20) | 0.28 | 122.69  (**3.95e^-05*^**) | 0.91(0.18, 4.56) | 0.91 | 0.92(0.56, 1.50) | 0.73 |
| Sleepless/  Insomnia | 1.02(0.69, 1.52) | 0.91 | 33.61  (0.67) | 0.84(0.26, 2.68) | 0.77 | 0.85(0.45, 1.62) | 0.63 |
| Total adiposity | | | | | | | |
| Body mass index | 1.27(1.15, 1.40) | **1.55e^-06^*** | 436.54  (**0.0005***) | 1.36(1.05, 1.75) | **0.02*** | 1.26(1.09,1.45) | **0.002*** |
| Body fat percentage | 1.25(1.10, 1.43) | **9.12e^-04^*** | 480.14  (**1.02e^-05^***) | 1.78(1.16, 2.72) | **0.009*** | 1.30(1.09, 1.55) | **0.004*** |
| Body fat distribution | | | | | | | |
| Waist circumference | 1.40(1.25, 1.58) | **9.02e^-09^*** | 439.87  (**7.49e^-05*^**) | 1.56(1.12, 2.16) | **0.008*** | 1.46(1.23, 1.72) | **1.06e^-05^*** |
| Waist-to-hip ratio | 1.11(1.00, 1.22) | **0.04*** | 407.41  (**5.68e^-05*^**) | 1.14(0.91, 1.43) | 0.27 | 1.09(0.93, 1.29) | 0.27 |
| Trunk fat mass | 1.21(1.11, 1.32) | **2.28e^-05^*** | 477.73  (0.002) | 1.25(0.97, 1.59) | 0.08 | 1.20(1.06, 1.36) | **0.005*** |
| Hip circumference | 1.16(1.05, 1.27) | **0.002*** | 520.35  (**1.05e^-05*^**) | 1.14(0.89, 1.47) | 0.30 | 1.17(1.03, 1.34) | **0.02*** |
| Leg fat mass (left) | 1.30(1.16, 1.46) | **4.04e^-06^*** | 487.00  (**0.002***) | 1.55(1.13, 2.13) | **0.007*** | 1.30(1.10, 1.54) | **0.002*** |
| Arm fat mass (left) | 1.26(1.15, 1.38) | **2.45e^-07^*** | 470.16  (**0.009***) | 1.29(1.01, 1.65) | **0.04*** | 1.24(1.09, 1.42) | **0.001*** |
| IVW, inverse‐variance weighted; OR, odds ratio; CI, confidence interval | | | | | | | |

**Table S3. Mendelian randomization estimates of the associations between lifestyle behaviors, and body fat distribution and risk of diabetic neuropathy**

| Exposure | IVW method | |  | MR-Egger | | Weighted Median | |
| --- | --- | --- | --- | --- | --- | --- | --- |
|  | OR (95%CI) | *P* Value | Cochran’s Q  (P Value) | OR (95%CI) | *P* Value | OR (95%CI) | *P* Value |
| Exercise | | | | | | | |
| Moderate to vigorous physical activity level | 0.41(0.04, 4.07) | 0.45 | 56.04  (**8.78e^-06^***) | 2.67e^-07^ (2.42e^-10^, 2.94e^-04^) | **5.58e^-04^*** | 0.65(0.10, 4.41) | 0.66 |
| Smoke | | | | | | | |
| Cigarettes per day | 1.31(0.89, 1.94) | 0.18 | 38.26  (**0.01***) | 1.28(0.63, 2.59) | 0.50 | 1.34(0.90, 1.99) | 0.15 |
| Alcohol intake | | | | | | | |
| Alcohol intake frequency | 1.06(0.70, 1.60) | 0.77 | 97.57  (0.27) | 0.27(0.08, 0.92) | **0.04*** | 0.78(0.42, 1.45) | 0.43 |
| Sleep | | | | | | | |
| Sleep duration | 0.41(0.16, 1.08) | 0.07 | 73.88  (0.26) | 0.06(0.001, 2.58) | 0.15 | 0.60 (0.15,2.44) | 0.47 |
| Sleepless/  Insomnia | 1.01(0.30, 3.37) | 0.99 | 33.94  (0.66) | 2.31(0.07, 79.42) | 0.64 | 1.06(0.16, 6.90) | 0.95 |
| Total adiposity | | | | | | | |
| Body mass index | 2.60(1.95, 3.45) | **5.77e^-11^*** | 408.97  (**0.009*)** | 5.03(2.41, 10.53) | **2.32e^-05^*** | 2.43(1.48, 3.98) | **4.39e^-04^*** |
| Body fat percentage | 2.53(1.74, 3.68) | **1.18e^-06^*** | 415.73  (**0.01***) | 2.04(0.61, 6.88) | 0.25 | 2.58(1.44, 4.60) | **0.001*** |
| Body fat distribution | | | | | | | |
| Waist circumference | 2.88(2.08, 3.99) | **1.73e^-10^*** | 377.38  (**0.05***) | 5.48(2.19,13.7) | **3.16e^-04^*** | 2.66(1.49, 4.73) | **8.94e^-04^*** |
| Waist-to-hip ratio | 1.42(1.09, 1.86) | **0.01*** | 338.68  (0.07) | 1.14(0.61, 2.14) | 0.68 | 1.05(0.67,1.63) | 0.84 |
| Trunk fat mass | 1.93(1.48, 2.52) | **1.23e^-06^*** | 481.77  (**0.002***) | 2.42(1.15, 5.12) | 0.02 | 2.35(1.56,3.52) | **3.96e^-05^*** |
| Hip circumference | 2.12(1.63, 2.77) | **2.85e^-08^*** | 466.69  (**0.005***) | 1.92(0.93, 3.97) | 0.08 | 2.39(1.58, 3.61) | **3.56e^-05^*** |
| Leg fat mass (left) | 2.65(1.91, 3.67) | **5.04e^-09^*** | 518.69  (**4.85e^-05*^**) | 7.90(3.15, 19.85) | **1.40e^-05^*** | 2.97(1.69, 5.22) | **1.49e^-04^*** |
| Arm fat mass (left) | 2.06(1.58, 2.69) | **1.17e^-07^*** | 469.30  (**0.01***) | 4.64(2.22, 9.71) | **5.49e^-05^*** | 2.33(1.47, 3.70) | **3.14e^-04^*** |
| IVW, inverse‐variance weighted; OR, odds ratio; CI, confidence interval | | | | | | | |

**Table S4. Statistical power of diabetic nephropathy**

| Exposure | OR | R^2^ | Power |
| --- | --- | --- | --- |
| Exercise | | | |
| Moderate to vigorous physical activity level | 0.12 | 0.001 | 0.37 |
| Smoke | | | |
| Cigarette per day | 1.03 | 0.03 | 0.06 |
| Alcohol intake | | | |
| Alcohol intake frequency | 0.99 | 0.02 | 0.07 |
| Sleep | | | |
| Sleep duration | 0.74 | 0.004 | 0.13 |
| Sleepless/Insomnia | 2.09 | 0.002 | 0.79 |
| Body fat distribution | | | |
| Body mass index | 1.92 | 0.04 | 0.94 |
| Body fat percentage | 1.85 | 0.03 | 1.00 |
| Waist circumference | 2.30 | 0.033 | 1.00 |
| Waist-to-hip ratio | 1.54 | 1.09e^-04^ | 1.00 |
| Trunk fat mass | 1.51 | 1.05e^-04^ | 1.00 |
| Hip circumference | 1.42 | 0.05 | 1.00 |
| Leg fat mass (left) | 2.28 | 0.03 | 1.00 |
| Arm fat mass (left) | 1.80 | 0.05 | 1.00 |

**Table S5. Statistical power of diabetic retinopathy**

| Exposure | OR | R^2^ | Power |
| --- | --- | --- | --- |
| Exercise | | | |
| Moderate to vigorous physical activity level | 0.36 | 0.002 | 0.96 |
| Smoke | | | |
| Cigarette per day | 1.16 | 0.03 | 0.91 |
| Alcohol intake | | | |
| Alcohol intake frequency | 1.05 | 0.02 | 0.15 |
| Sleep | | | |
| Sleep duration | 0.80 | 0.004 | 0.34 |
| Sleepless/Insomnia | 1.02 | 0.002 | 0.05 |
| Body fat distribution | | | |
| Body mass index | 1.27 | 0.05 | 0.94 |
| Body fat percentage | 1.25 | 0.03 | 1.00 |
| Waist circumference | 1.40 | 0.033 | 1.00 |
| Waist-to-hip ratio | 1.11 | 0.05 | 0.84 |
| Trunk fat mass | 1.21 | 0.05 | 1.00 |
| Hip circumference | 1.15 | 0.03 | 0.98 |
| Leg fat mass (left) | 1.30 | 0.03 | 0.87 |
| Arm fat mass (left) | 1.26 | 0.05 | 1.00 |

**Table S6. Statistical power of diabetic neuropathy**

| Exposure | OR | R^2^ | Power |
| --- | --- | --- | --- |
| Exercise | | | |
| Moderate to vigorous physical activity level | 0.41 | 0.002 | 0.17 |
| Smoke | | | |
| Cigarette per day | 1.03 | 0.04 | 0.06 |
| Alcohol intake | | | |
| Alcohol intake frequency | 1.06 | 0.023 | 0.06 |
| Sleep | | | |
| Sleep duration; | 0.41 | 0.004 | 0.30 |
| Sleepless/Insomnia | 1.01 | 0.002 | 0.05 |
| Body fat distribution | | | |
| Body mass index | 2.60 | 0.05 | 1.00 |
| Body fat percentage | 2.53 | 0.03 | 1.00 |
| Waist circumference | 2.88 | 0.03 | 1.00 |
| Waist-to-hip ratio | 1.42 | 0.05 | 0.95 |
| Trunk fat mass | 1.93 | 0.05 | 1.00 |
| Hip circumference | 2.12 | 0.05 | 1.00 |
| Leg fat mass (left) | 2.65 | 0.03 | 1.00 |
| Arm fat mass (left) | 2.06 | 0.05 | 1.00 |

**Figure S1. Funnel plots of diabetic nephropathy**


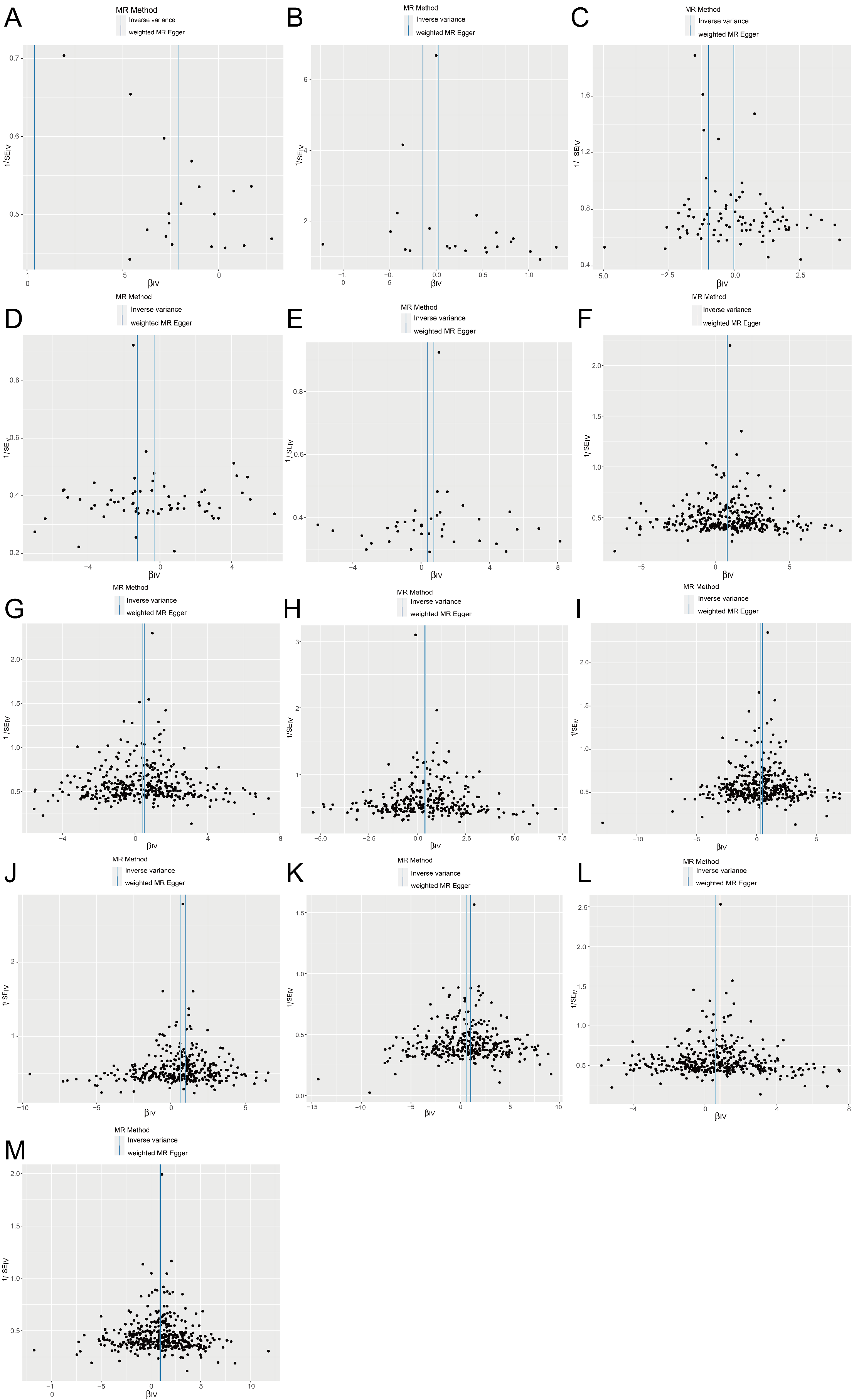


A. Moderate to vigorous physical activity level; B. Cigarette per day; C. Alcohol intake frequency; D. Sleep duration; E. Sleepless/Insomnia; F. Waist circumference G. Trunk fat mass; H. Waist-to-hip ratio; I. Hip circumference; J. Body mass index; K. Body fat percentage; L. Arm fat mass (left); M. Leg fat mass (left)

**Figure S2. Funnel plots of diabetic retinopathy**


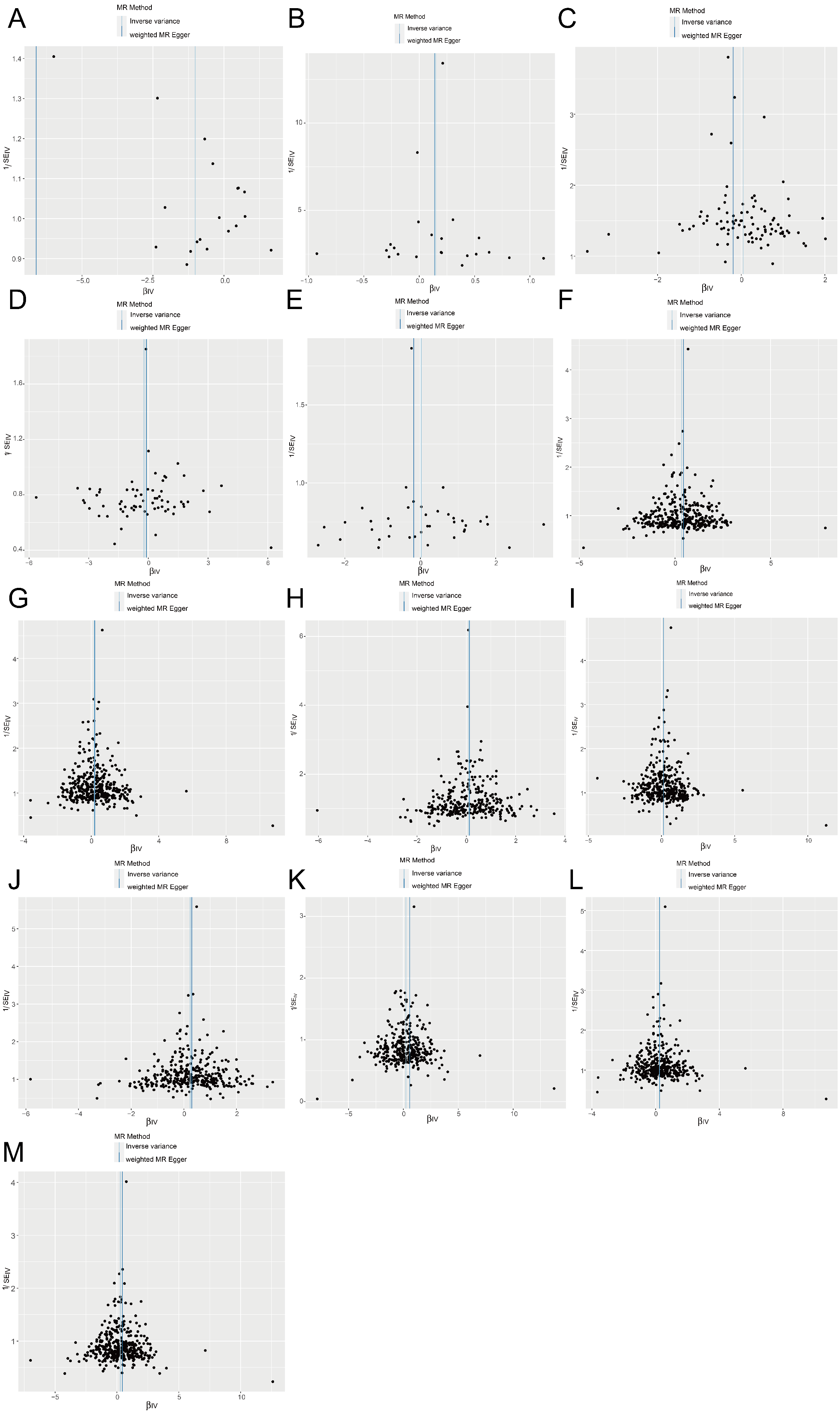


A. Moderate to vigorous physical activity level; B. Cigarette per day; C. Alcohol intake frequency; D. Sleep duration; E. Sleepless/Insomnia; F. Waist circumference G. Trunk fat mass; H. Waist-to-hip ratio; I. Hip circumference; J. Body mass index; K. Body fat percentage; L. Arm fat mass (left); M. Leg fat mass (left)

**Figure S3. Funnel plots of diabetic neuropathy**


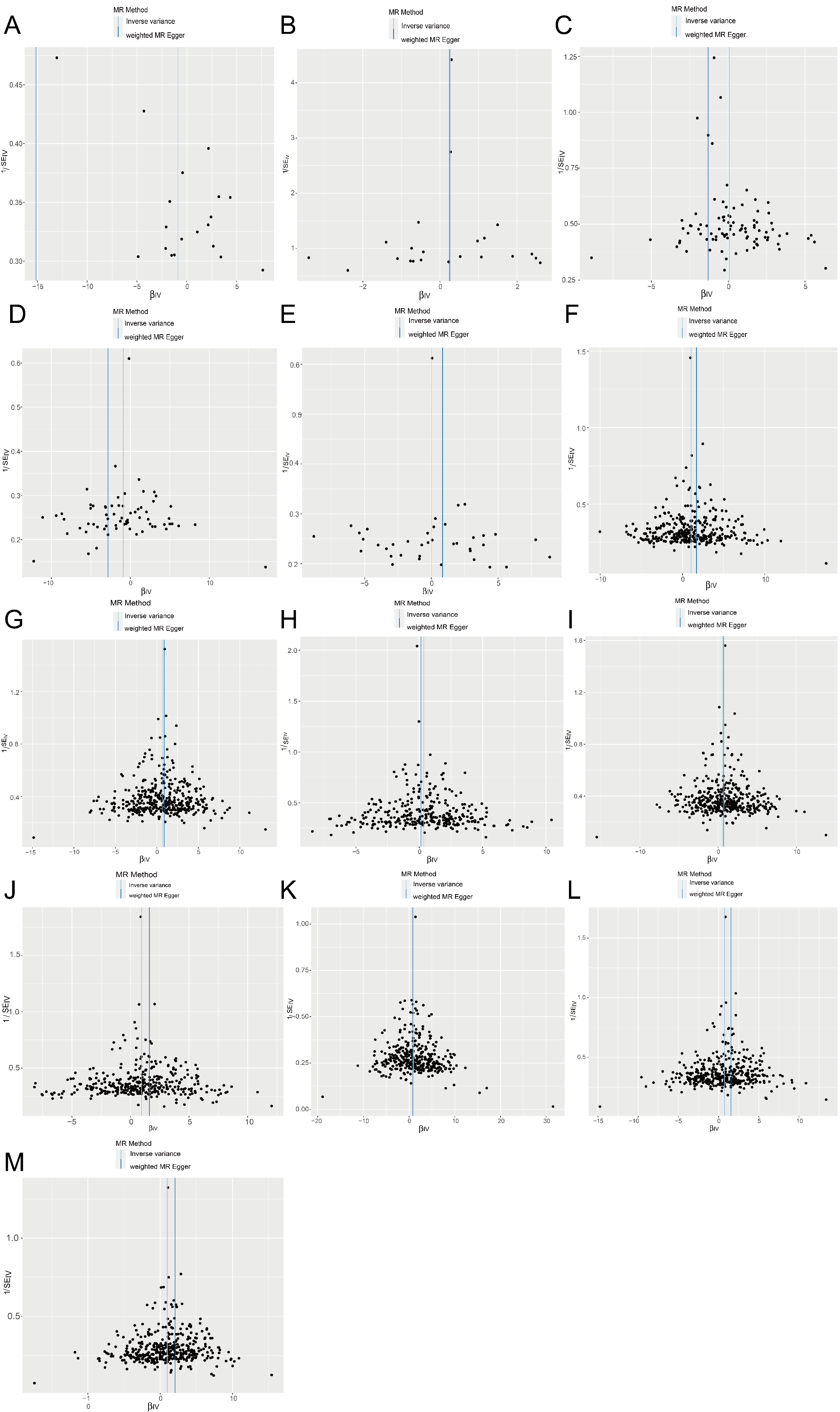


A. Moderate to vigorous physical activity level; B. Cigarette per day; C. Alcohol intake frequency; D. Sleep duration; E. Sleepless/Insomnia; F. Waist circumference G. Trunk fat mass; H. Waist-to-hip ratio; I. Hip circumference; J. Body mass index; K. Body fat percentage; L. Arm fat mass (left); M. Leg fat mass (left)

**Figure S4. MR-Egger intercept scatter plots of diabetic nephropathy**


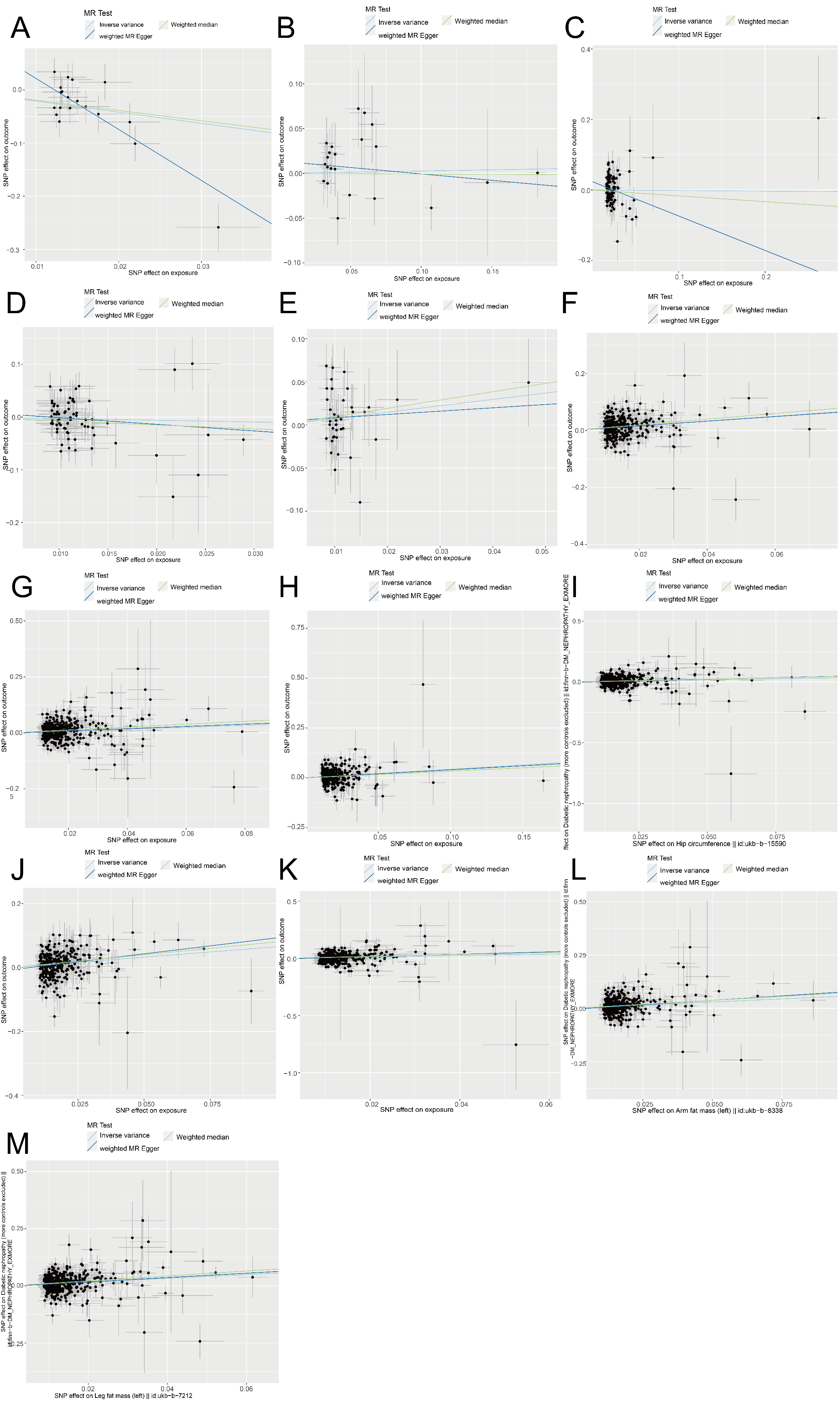


A. Moderate to vigorous physical activity level; B. Cigarette per day; C. Alcohol intake frequency; D. Sleep duration; E. Sleepless/Insomnia; F. Waist circumference G. Trunk fat mass; H. Waist-to-hip ratio; I. Hip circumference; J. Body mass index; K. Body fat percentage; L. Arm fat mass (left); M. Leg fat mass (left)

**Figure S5.MR-Egger intercept scatter plots of diabetic retinopathy**


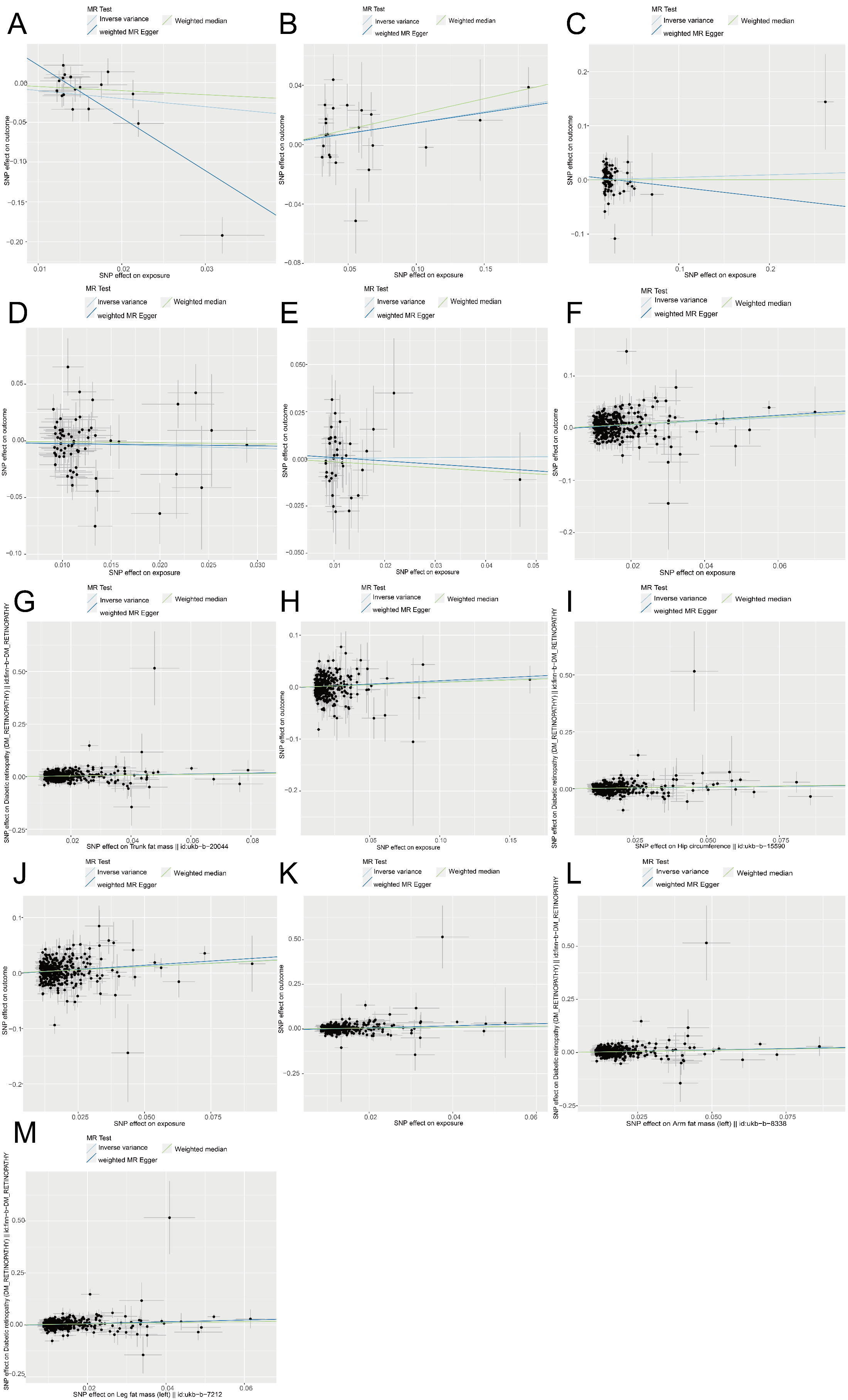


A. Moderate to vigorous physical activity level; B. Cigarette per day; C. Alcohol intake frequency; D. Sleep duration; E. Sleepless/Insomnia; F. Waist circumference G. Trunk fat mass; H. Waist-to-hip ratio; I. Hip circumference; J. Body mass index; K. Body fat percentage; L. Arm fat mass (left); M. Leg fat mass (left)

**Figure S6.MR-Egger intercept scatter plots of diabetic retinopathy**


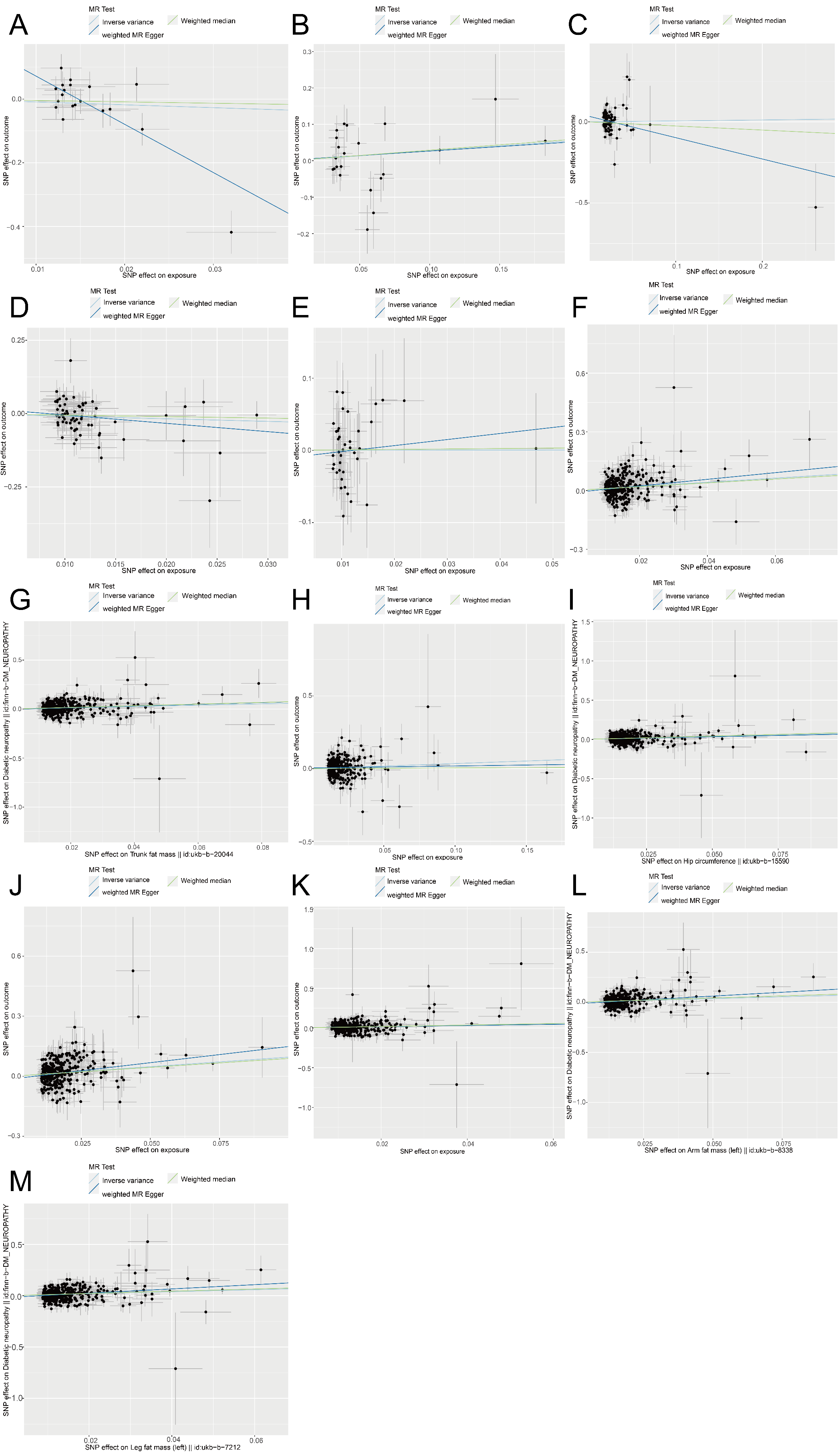


A. Moderate to vigorous physical activity level; B. Cigarette per day; C. Alcohol intake frequency; D. Sleep duration; E. Sleepless/Insomnia; F. Waist circumference G. Trunk fat mass; H. Waist-to-hip ratio; I. Hip circumference; J. Body mass index; K. Body fat percentage; L. Arm fat mass (left); M. Leg fat mass (left)
